# Emergence of National Nutrition Policy in the Lao People’s Democratic Republic: an analysis of collaborations between governmental and external actors

**DOI:** 10.1186/s41182-023-00532-w

**Published:** 2023-08-08

**Authors:** Viengsamay Sengchaleun, Sengchanh Kounnavong, Daniel Reinharz

**Affiliations:** 1 Department of Social and Preventive Medicine, Laval University, Quebec, QC Canada; 2 Lao Tropical and Public Health Institute, Ministry of Health, Vientiane, Lao PDR

**Keywords:** Malnutrition, Lao PDR, Multisectoral collaborations, Stakeholders, Coalition, Nutrition policy

## Abstract

**Background:**

In most developing countries, addressing malnutrition involves a coalition of stakeholders that includes the government and international development partners. This study explores the evolution of the malnutrition actor coalition landscape before and after the emergence of the National Nutrition Policy in the Lao People’s Democratic Republic (Lao PDR) in 2008.

**Methods:**

A qualitative study was conducted based on the theory of coalition structuring. Twenty semi-structured interviews were performed with representatives of national and international organisations involved in addressing malnutrition in Lao PDR. The information obtained from the interviews was complemented by an analysis of relevant documents dating back to 1990. Interviews were recorded and transcribed verbatim. A thematic analysis was performed using NVivo 11 software and the diagrams of collaboration drawn by the participants were turned into a visual collaboration map using SocNetV software. We relied on various types of triangulation to increase the analysis's credibility, reliability, and confirmability.

**Results:**

The results showed that before the emergence of the National Nutrition Policy, three coalitions representing the health, agriculture, and education sectors coexisted. These colalitions worked largely in silos, although with some interactions when deemed necessary mainly by United Nations agencies. The emergence of the National Nutrition Policy provided the government with an effective political tool for coalescing the three coalitions into a unique coalition involving all major stakeholders in the nutrition field. All three forces that incite actors to collaborate inside a coalition according to the theory of coalition structuring (transactions, control, intangible factors) were mobilised in the creation of the single coalition.

**Conclusions:**

Combating malnutrition is a government priority in the Lao PDR. The current study showed that the National Nutrition Policy in Lao PDR has led to a significant evolution in the malnutrition coalition landscape, resulting in improved collaboration among stakeholders. This finding highlights the effectiveness of public policies in facilitating intersectoral activities to tackle complex problems, such as malnutrition.

## Introduction

The World Health Organisation (WHO) defines malnutrition as deficiencies, excesses, or imbalances in a person’s intake of energy and/or nutrients. Malnutrition covers three groups of conditions: (1) undernutrition (deficiency of calories or nutrients to meet an individual's needs to maintain good health); (2) micronutrient-related malnutrition, which includes micronutrient deficiencies or micronutrient excess; and (3) overweight and obesity [1]. In this article, malnutrition refers to undernutrition and micronutrient deficiencies.

Malnutrition is a determinant of vulnerability to disease and death. Approximately 45% of all deaths among children under 5 years are linked to malnutrition [2].

The Lao People’s Democratic Republic (Lao PDR), or Laos, is a developing country with a high malnutrition rate. In 2017, stunting was estimated to affect 33% of Lao children under 5 years of age. Underweight, meanwhile, affects 21% of children. These rates are higher than those of neighbouring countries. While chronic malnutrition affects a larger proportion of the population, acute malnutrition, as indicated by a 9% prevalence of wasting in children under five, remains a significant concern [3].

In the 1990s, the government of Lao PDR, in collaboration with international development partners, i.e., nongovernmental organisations (NGOs), United Nations (UN) agencies, and bi- and multilateral cooperation, started to implement interventions to reduce the number of children impacted by malnutrition. Some of these efforts were aimed at addressing poverty, boosting agricultural productivity, and improving healthcare infrastructure. However, despite the efforts made to address malnutrition in Lao PDR during the 1990s and 2000s, progress was limited. Malnutrition remained at a similarly high prevalence among children under 5 years of age, and, in some rural areas, even increased [4]. In 1993, the first National Health Survey documented a prevalence of 48% of stunting in children under 5 years of age [5]. In 2006, the Lao Multiple Indicator Cluster Survey showed that stunting, underweight, and wasting affected 40%, 37%, and 7% of children under 5 years, respectively [6]. In the 1990s and 2000s, Lao PDR was classified by the WHO as a country with a very high prevalence of malnutrition (defined as a stunting prevalence in children under 5 years ≥ 40%), greater than the Southeast Asian countries’ average [6–8]. An overhaul of addressing malnutrition was obviously necessary. In 2008, the government introduced the National Nutrition Policy (NNP) as a tool to dramatically change how Lao PDR addressed malnutrition [9].

Before the emergence of the NNP, the lack of cooperation between the concerned sectors was seen as the main determinant of the relative ineffectiveness of nutrition activities in Lao PDR. The diagnosis was that the lack of cooperation resulted from the absence of a single coalition of actors concerned with malnutrition [10]. A coalition can be defined as a group or organisations that might belong to different sectors of the society and that agree to work together to achieve a common objective [11]. To build this coalition, it was then proposed to develop a new public policy. A public policy was seen as a promising vehicle for bringing organisations from different sectors together to collaborate with each other [9]. The NNP is a legally binding document that led to the formal integration of nutrition into the National Socio-economic Development Plans in line with the implementation of the National Growth and Poverty Eradication Strategy [9, 12]. The NNP called for more effective cooperation between domestic and external actors in all sectors, such as the health, agriculture, education, and social development sectors [9].

The literature in the field of organisations supports this proposition. In the field of health promotion, building coalitions of stakeholders who can be extremely diverse (governments, nonprofit agencies, the private sector, universities, and interested citizens) is an effective strategy that is often used to support the emergence of multisectoral policies on different topics, such as food labelling, or tobacco consumption [11, 13]. Coalitions help actors coming from different sectors to overcome divergent interests to collaborate to achieve a common goal [11, 14].

The emergence of a national policy can have a significant impact on the coalition landscape and the implementation of strategies to combat malnutrition. A national policy provides a framework for action and sets clear goals and objectives for addressing malnutrition. This policy creates opportunities for the collaboration of different stakeholders to share ideas and resources and coordinate their efforts, towards a common goal, as well as to obtain more support, funding, and resources for addressing malnutrition. A policy shows that its objectives are a priority for the government. Above all, policies serve as a tool for legitimising the actions that the government will support [9]. This study aimed to assess the impact of the NNP on the fight against malnutrition in Lao PDR. Specifically, the objective of this study was to explore the evolution of the coalition of actors involved in the field of malnutrition in the Lao PDR, before and after the emergence of the country's National Nutrition Policy in 2008. Understanding the evolution of coalitions before and after the policy's implementation can help determine how the policy has led to more effective and coordinated efforts to address malnutrition. Examining the evolution of coalitions can help identify the role played by different stakeholders, such as government agencies, development partners, and nongovernmental organisations, in addressing malnutrition. These findings can be used to inform decision-makers at multiple levels to strengthen ongoing and future programming design, monitoring, and evaluation to promote vertical and horizontal coordination among different sectors to reduce malnutrition as well as tackle other health-related issues in Lao PDR.

## Methods

### Conceptual framework

The theory of coalition structuring by Gamson, reviewed by Lemieux (1998), was used to analyse the coalition dynamics of actors concerned with malnutrition in Lao PDR [15].

There are several theories that allow to study the dynamic of coalitions. Each theory brings its specific lens to the interpretation of the key role of coalitions in supporting the emergence, implementation, and functioning of a public policy. For example, Advocacy Coalition Framework emphasises the role of beliefs and values in coalition building [16]. Policy Network Analysis focuses on the network of relationships between actors involved in a coalition, assuming that an actor's position in society, their skills and resources, external factors, and their interactions with other actors can all impact the policy process and its outcomes [17].

The theory of coalition structuring that we used, seemed to us particularly relevant, based on the literature that shows that this approach is an effective tool for revealing dynamics underpinning the emergence of policies [18]. This theory provides a framework for understanding how different actors come together to achieve their policy goals, how power and resources are distributed among these actors, and how they navigate the challenges and conflicts that arise in the policy-making process [15]. These aspects were intuitively felt to be present in the case of the nutrition policy in Lao PDR. This theory has been applied by researchers to interpret coalitions and their impact on the evolution of health care policies, programs, or interventions, including in the field of nutrition [18, 19]. The theory of coalition structuring proposes three reasons for actors to join a coalition: transactions, intangible factors, and control. Transactions refer to the fact that actors bring with them different resources and that joining a coalition allows them to exchange these resources for an expected benefit that they would not be able to obtain if they worked alone or within another coalition. Intangible factors relate to the emotional aspects carried by a shared ideology or the fact of knowing and appreciating each other, which push these actors to want to work together. Control relates to a power structure that is used to force actors to participate in a coalition [15].

### Data collection

The methodological approach was qualitative and used a case study to explore in depth a subject that had been hitherto unexplored in Lao PDR.

The study took place in Vientiane capital, between March and June 2020. The main data source was semi-structured interviews, which was complemented with relevant documents.

Semi-structured interviews were conducted with government and external actors who were in a high-level position related to the emergence or implementation of the nutrition policy in Lao PDR. In this study, external actors refer to nongovernmental actors, especially foreign actors (foreign NGOs, United Nations agencies and bi- and multilateral cooperation). These actors can be particularly decisive in encouraging the development of a policy and marking it with a given orientation. The list included national policy advisors, high-ranked public servants in public organisations of the health sector involved in nutrition, and managers of NGOs. The choice of the participants was made based on four criteria: (1) the involvement of their organisation in addressing malnutrition; (2) their position in the organisation having a tenure of at least 1 year, allowing them to understand the activities deployed in connection with the health of the populations; (3) their knowledge and experience about the involvement of their organisation in addressing malnutrition before and after the emergence of the National Nutrition Policy; and (4) the fact that their participation would increase the diversity of opinion in the current study. Organisations that should be included in the sample were purposely identified with the help of the Lao Tropical and Public Health Institute and National Nutrition Centre. These Institutes retain the main expertise in research and interventions in the nutrition field in the country. The initial sample was completed using the snowball sampling approach [20].

Twenty semi-structured interviews were conducted. Six participants were high-ranked public servants, four were policy advisors, three were NGO directors, six were case managers, and one was an external consultant. Eight participants did not respond to our solicitation (one former minister, two managers of a government organisation, and five external organisations’ managers). The characteristics of the participants (P) are shown in Table 1.

**Table 1:** Basic characteristics of the participants

Age (years)	
Mean	44
Minimum–maximum	33–63
Gender (*n* = 20)	
Male	6
Female	14
Mean amount of time the respondent has been in their job (years)	8
Number of respondents who were in their agency prior to 2008 (*n* = 20)	7
Organisations of the participants (*n* = 20)	
Government (ministries or institutions belonging to a ministry)	12
UN agencies	3
NGOs	3
Bi/multilateral cooperation	1
Consultancies^*^	1
Preferred language (*n* = 20)	
Lao	17
English	2
French	1

*n* number of participants

^*^External individuals who are hired for a particular job that cannot be done by someone in the organisation

Interviews consisted of face-to-face meetings, video conferences, and phone meetings. They were conducted in Lao, English, or French, lasted between 45 and 90 min, and were audio-recorded with the consent of the participants. During and after the interviews, handwritten notes were taken.

The interview guide used to conduct the exchanges was based on the theory of coalition structuring. The topics for discussion related to the theory's three factors (i.e., transactions, intangible factors, and control) that influence coalition formation. They also provided an opportunity to initiate discussions on the perceived role of coalitions in the evolution of addressing malnutrition in the country. To facilitate the exchanges, participants were encouraged to support their comments by drawing a diagram that visually presented their perception of the organisations involved and the relationships between them both before and after the emergence of the National Nutrition Policy.

The interviews were first transcribed in the language spoken during the interviews. The interviews in Lao were thereafter translated into French. As soon as a transcript was produced, it was sent to another researcher and discussed to explore whether any aspects should be examined in greater depth in subsequent interviews.

In addition to the interviews, documents written in English, Lao, or French dating back to 1990 were analysed. These documents were documents received from the respondents and documents available on the websites of their organisations. We also performed additional searches in *Google Scholar* using combinations of the keywords: “Nutrition policy”, “Laos”, “Lao PDR”, “Malnutrition”, “Undernutrition”, “Government”, “NGO”, “United Nations”, “International Agencies”, “Coalition”, “Collaboration”, “Cooperation”, “Alliance”, and “Partnership”.

In total, nine documents were examined, including reports, national surveys, and national plans and strategies that indicated how nutrition was addressed before and after the emergence of the National Nutrition Policy in Lao PDR.

### Data analysis

The interviews and documents were analysed using NVivo 11 software (qualitative data analysis software, QSR International Pty Ltd., Australia).

We performed a thematic analysis [21, 22]. The analyses were based on an inductive–deductive approach [21, 23]. Data coding was performed by one researcher and was validated by another researcher throughout the analysis process. When a disagreement arose or if an external point of view was needed, the third researcher was consulted.

Coding was based on the theory of coalition structuring. We focused on the three dimensions that shape the structure of a coalition, i.e., transactions, intangible factors, and control.

The diagrams of collaboration drawn by the participants were turned into a visual collaboration map using SocNetV software. Integration of the diagrams was complemented with information that was either provided orally or obtained from the documents provided by the participants. The final diagram aimed to present the characteristics and intensity of the collaboration between the actors concerned with malnutrition in Lao PDR.

The credibility, reliability, and confirmability of the analysis were based on different types of triangulation, including triangulation of methods (using two different data collection methods), triangulation of sources (including participants from various sectors/positions), and triangulation of analysts (interview coding and data interpretation were validated by an additional one or two researchers) to minimise the prejudices, values, and ideologies of the researcher that might have influenced the interpretation of the data [21, 24].

## Results

The following sections present the evolution of the coalition of actors concerned with malnutrition in Lao PDR under the emergence of the National Nutrition Policy.

### Coalitions of actors before the emergence of the National Nutrition Policy

Since the 1990s, one of the fundamental demands of the Lao government to its ministries has been that they define priorities and strategies that allow them to contribute to a central objective, which is to reduce poverty in the country [25, 26].

As a result, nutrition has become part of the country's developmental plan. A well-nourished population is more productive, which will support the development of the country, hence the diminution of its poverty rate [26]. Three sectors were particularly involved in activities aimed at improving the nutritional status of the population: health, agriculture, and education. Each of these sectors had its own coalition of actors.

**Fig. 1:**
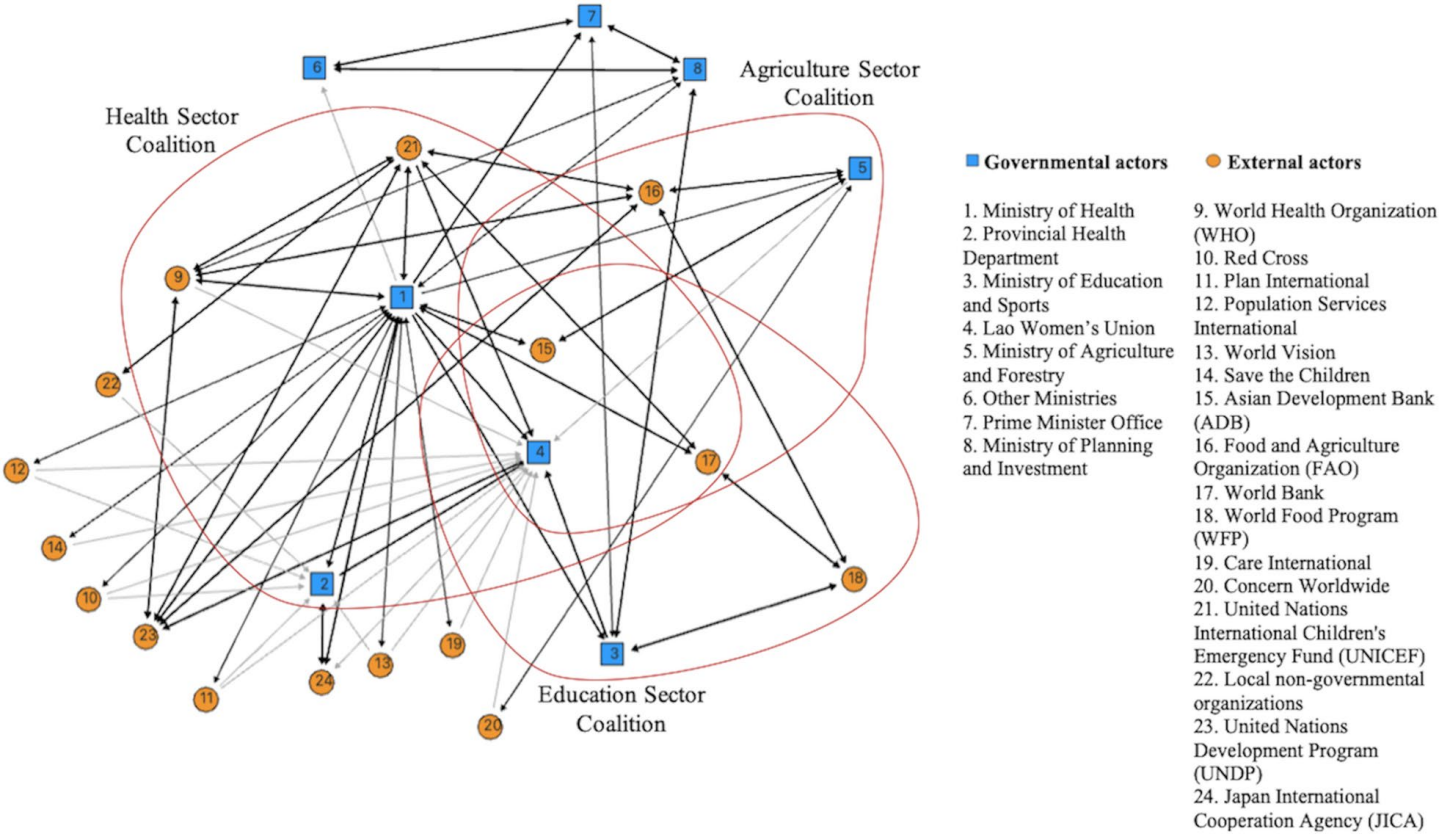
Coalition landscape before the emergence of the National Nutrition Policy. The circles indicate coalition members. Unidirectional arrows show a collaboration aimed at the pursuit of the self-interests of the member at the arrow's origin. Bidirectional arrows show collaboration resulting from a negotiation between both sides on how to achieve a common goal. The intensity of the collaboration is indicated by black lines, representing a collaboration mentioned by both collaborators as a process of exclusive exchanges that led to a satisfying outcome for both of them. Grey lines represent a collaboration based on several meetings between both organisations, alone or with others, without being mentioned as a special and intense relationship between both partners

Before the emergence of the NNP in 2008, there was little collaboration between these coalitions. The coalitions worked largely in silos. Where collaborations did exist, they were often initiated by an external organisation, notably UN agencies. These organisations that worked with different sectors occasionally saw the value of building bridges between their projects in different sectors (Fig. 1).

#### Coalition dimension before the emergence of the National Nutrition Policy

Prior to the adoption of the National Nutrition Policy in 2008, there was little literature on the value of multisectoral interventions related to nutrition issues in Lao PDR. Only the health sector made nutrition a priority. Yet, in the Lao health sector, interventional approaches to nutrition were limited, according to the majority of respondents (16 of 20 respondents). The interventions were essentially confined to prevention activities based on health education programs that were traditionally carried out in mother-and-child health improvement campaigns [5]. In non-health sectors, such as agriculture and education, malnutrition reduction was considered as an expected side benefit of their interventions rather than the main driver of action [10].

According to 11 respondents who represented government institutions, the inter-coalition partnership essentially involved the Ministry of Health and the Ministry of Education and Sports. Both ministries have been collaborating to formulate the first National School Health Policy in 2005 [27]. However, partnerships between the two sectors were often limited to specific interventions, such as school deworming campaigns, vitamin A supplementation campaigns, or providing nutritious school lunches. Transactions mainly consisted of the exchange of expertise in prevention programmes for access to the target population of children. These transactions were reinforced by intangible factors. Coalitions were mainly driven by the ministries, which tended to work with external organisations with which they had previous experience collaborating. This collaborative experience had enabled them to determine a convergent vision of the actions to be taken. Notably, the control factor appeared to play a minimal role in the interactions between coalitions. According to 12 respondents, this particularly occurs when there is no possibility for an organisation to legitimately imposing its will.

*“In the past, malnutrition was known as the issue of the health sector… collaboration between sectors was occasionally and only for certain programmes that required the involvement of other sectors, for example, the school deworming programme, launched by Ministry of Health and Ministry of Education”.* [P1]

The following sections will successively present the characteristics of the three coalitions. The results of the analysis of the conceptual framework’s dimensions are presented in Table 2.

**Table 2:** Coalition dimension before the emergence of the National Nutrition Policy

Coalitions		Health sector coalition	Agriculture sector coalition	Education sector coalition
Leader		Ministry of Health	Ministry of Agriculture and Forestry	Ministry of Education and Sport
Main objective		Decrease in malnutrition	Food security	Decrease in absenteeism and increase school enrolment rate
Intracoalition transactions	Assets (government)	• Provider of legitimacy on health- and nutrition-related issues • Provider of authorisation to access target-populations	Provider of legitimacy on food security issues	Provider of authorisation to access target-populations
	Expected benefits (government)	Financial and technical support	Financial and technical support	Financial and technical support
	Assets (development partners)	• Funds • Technical expertise	• Funds • Technical expertise	• Funds • Technical expertise
	Expected benefits (development partners)	Fulfilling mandates to address health concerns in the country	Fulfilling mandates to address health concerns in the country	Fulfilling mandates to address health concerns in the country
Intracoalition intangible factors		• Having previous collaborations • Interpersonal relationships	• Having previous collaborations • Interpersonal relationships	• Having previous collaborations • Interpersonal relationships
Intracoalition control		Control by the ministry over the budget	Control by the ministry over the budget	Control by the ministry over the budget

Table 2 presents the three coalitions, their leaders, and the main objective pursued by the coalitions in relation to nutrition, as well as the conceptual framework’s dimensions (transactions, intangible factors, and control).

*Health sector coalition* The health sector coalition consisted of its leader, the Ministry of Health (Department of Hygiene and Health Promotion and Mother and Child Health Centre), the Lao Women’s Union, the WHO, the United Nations International Children's Emergency Fund (UNICEF), the Asian Development Bank, and the World Bank (Fig. 1).

The relationship between the members of the health sector coalition was based, above all, on transactions (Table 2). The transactions supported nutrition-related activities, notably universal salt iodisation, vitamin A and iron distribution, deworming programmes for children, the treatment of severe acute malnutrition, and breastfeeding promotion [5]. Transactions involved mainly the Ministry of Health on one hand and external actors on the other hand. The Ministry of Health’s role was to legitimise the implementation of activities provided by external actors. It also supplied human resources to perform outreach activities for nutrition interventions outside health care facilities, although these activities were under the supervision of provincial and district authorities. External actors contributed to the coalition by providing financial and technical support for performing nutrition interventions (e.g., national micronutrient and deworming campaigns, hygiene education for primary school children, etc.), capacity building, development guidelines and standards, research, monitoring, and nutrition intervention evaluations.

Several respondents (13 respondents) stressed that the mobilising effect of the transactions was reinforced by intangible factors. Intangible factors resulted from the long experience of collaboration between the Ministry of Health, UNICEF, and the WHO in multiple projects aiming to improve child and maternal health through nutrition interventions.

*Agriculture sector coalition* The agriculture sector coalition brought together the Ministry of Agriculture and Forestry (Department of Agriculture and Department of Livestock and Fisheries) as its leader, the Lao Women’s Union, the Asian Development Bank, the World Bank, and the Food and Agriculture Organisation (FAO) (Fig. 1). Reducing the number of individuals living under the food poverty line (defined as those who live with less than the daily minimum dietary energy requirements of 2100 kcal) was one of the top priorities of the Ministry. Therefore, the sector focused on increasing food production (e.g., rice, sugarcane, peanuts, corn, cassava, soybeans, fish, and livestock) and income generation. There were also investments in the construction of irrigation schemes to support the capacity of the population to reach food production targets.

Several participants stated that in this coalition, actors had been working together for a long time, which was the element that led to the creation of convergent views on the activities to be performed. Intangible factors were, therefore, strong. However, transactions played a decisive role, mainly based on the need to legitimise the external actors' activities in exchange for financing, material, and technical support.

*Education sector coalition* The Ministry of Education and Sports was the leader of this coalition, with the Lao Women’s Union, the World Food Programme, the Asian Development Bank, and the World Bank formed the education sector coalition (Fig. 1). This coalition aimed primarily to increase school enrolment and to encourage Lao children to complete their primary education. Among activities related to nutrition, the coalition implemented school feeding programs as well as hygiene and sanitation activities, such as hand washing, tooth brushing, and deworming activities twice per year [26]. The health-related activities aimed to create the health conditions that allow children to go to and succeed in school.

Transactions were the main cohesive factor of the coalition. International organisations provided food assistance, technical support, and financial resources. On the other hand, the Ministry of Education and Sports prepared the necessary authorisation documents and provided the on-the-ground human resources needed by external organisations to make their projects happen. Transactions were strengthened by intangible factors. The representatives of the organisations that were members of the coalition tended to know each other well, as most of them had a long history of collaboration.

With regard to the "control" factor, we note that a few participants (three participants) stated that control over the budgets by the ministries forced the actors to engage in collaboration. Yet, the participants stressed that, generally speaking, ministries were incited to use this tool by development partners.

### Coalition of actors after the emergence of the National Nutrition Policy

**Fig. 2:**
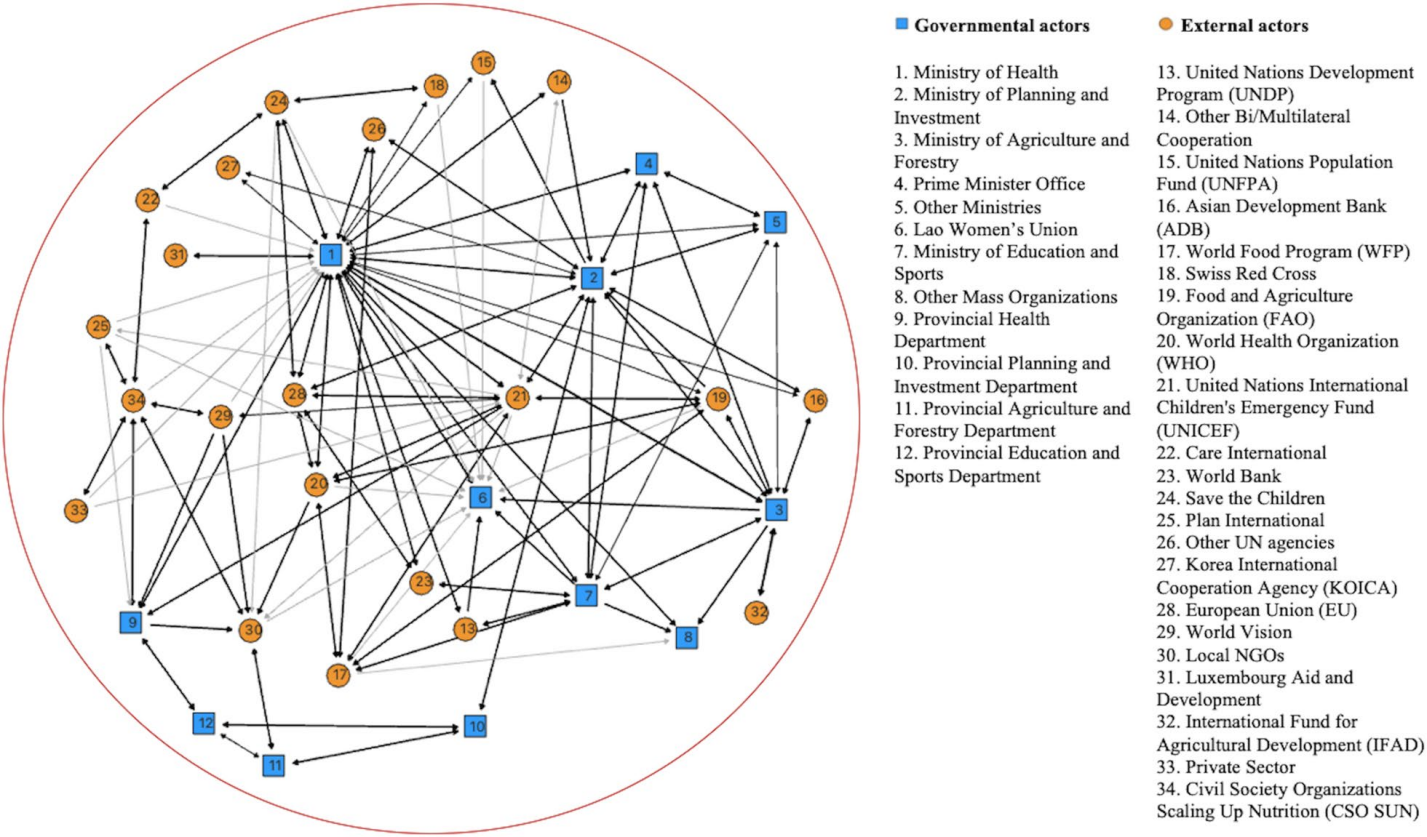
Coalition landscape after the emergence of the National Nutrition Policy. The circle indicates coalition members. Unidirectional arrows show a collaboration aimed at the pursuit of the self-interests of the member at the arrow's origin. Bidirectional arrows show collaboration resulting from a negotiation between both sides on how to achieve a common goal. The intensity of the collaboration is indicated by black lines, representing a collaboration mentioned by both collaborators as a process of exclusive exchanges that led to a satisfying outcome for both of them. Grey lines represent a collaboration based on several meetings between both organisations, alone or with others, without being mentioned as a special and intense relationship between both partners

After the emergence of the NNP in 2008, a major change took place in the landscape of coalitions. The existing coalitions merged into a large coalition involving all major stakeholders in the field of nutrition (Fig. 2).

#### Coalition dimension after the emergence of the National Nutrition Policy

The merger of three coalitions into a single coalition that brings together all the major actors lies in the three theoretical forces that support a coalition (transactions, intangible factors, and control), as presented in Table 3.

**Table 3:** Coalition dimension after the emergence of the National Nutrition Policy

Coalition leader		Ministry of Health			
Coalition members		Government	Development partners		
			UN agencies	Bi/multilateral cooperation	NGOs
Transactions	Assets	• Provider of authorisation to external actor to conduct activities • Provider of local human resources for collaborators • Providing the legitimised coordination between coalition members	• Experience • Funds • Technical expertise	• Funds • Technical expertise	• Experience • Technical expertise
	Expected benefits	• Training of government staff • Technical and financial support	• Fulfilling mandates • Human resources for implementing the activities	• Fulfilling mandates	• Budget • Access to target populations
Intangible factors		• Interpersonal relationship • One-party state ideology			
Control		• Formal delegation of leadership by the Prime Minister to the Minister of Health			

Table 3 presents the leader, the members of the coalition (government actors and development partners), and the factors (transactions, intangible factors, control) that brought these actors into the coalition.

The transactions mentioned above can be found in the new coalition. Seven respondents mentioned that their importance in keeping the coalition together is strengthened by the fact that there are more and more calls for proposals from funding agencies for intersectoral projects.

The single coalition also benefits from the intangible factors produced by a one-party state. The presence of a single party favours the convergence of interpretations of problems and solutions. Having a common ideology makes it easier to understand priorities and how to address priority needs. This creates a fertile ground for the execution of government orders to develop intersectoral projects.

However, the vast majority of respondents (17 respondents), including those who had been working in nutrition prior to 2008 and those who had not, agreed that the control factor had the greatest impact on the creation of a single functional coalition. Control was essentially provided by the National Nutrition Policy. This policy formally sanctioned the delegation of authority over nutrition by the Office of the Prime Minister to the Minister of Health. Under the authority of the Prime Minister, the Ministry of Health was empowered to bring together the complementary sectors (Table 3).

*“Because we have the policy and the strategy as a guide for implementing the interventions, and also the objectives had been defined…we are now applying the multisectoral approach to reduce malnutrition, all sectors are obliged to work on nutrition together for the same objective, to improve the nutritional status.”* [P8]

#### A single and effective coalition

The enactment of the NNP in 2008 brought major changes to the nutrition coalition landscape. A system that had been scattered coalesced into a single large coalition (Fig. 2). Members of the previous coalitions adopted one main common objective, namely, to make available to coalition members the assets that one member has but the others lacks [23].

All the respondents acknowledged the importance of the role of leader conferred to the Ministry of Health. Indeed, the Ministry of Health was formally designated to be the national leader of the nutrition coalition. This role was entrusted to the Ministry of Health by the National Council of the Office of the Prime Minister, which predicted that such an official endorsement would facilitate the implementation of collaborations with other ministries concerned [9]. The Ministry of Health was entitled to put in place coordination mechanisms with other ministries and concerned organisations, as well as local authorities at all levels. The Ministry of Health had to provide guidance, propose modifications to legislation, and design strategies, action plans, and regulatory measures for the effective implementation of the NNP; furthermore, the members of the coalition were active partners in the activities related to the priorities defined [29, 30].

The coalition is composed of several groups. The first group encompasses government organisations. At the national level, it consists of the Ministry of Health as the lead body along with the Ministries of Agriculture and Forestry, Education and Sports, and Planning and Investment. It also includes other relevant ministries and mass organisations. At the subnational level, it includes the provincial and district health, education, and agriculture offices. The second group consists of development partners, mainly the European Union and four UN agencies, i.e., the WHO, FAO, UNICEF, and the World Food Program, as well as the World Bank. These organisations are joined by other members, mainly other UN agencies, embassies, and cooperation agencies. The last group is formed by NGOs working in various sectors, including health, education, and agriculture; water, sanitation, and hygiene; capacity building; the environment; gender; economic empowerment; and labour and social welfare.

There was a consensus among the respondents that the large coalition is functional. All respondents affirmed that putting in place a large coalition that would merged the three existing ones was a necessary step to incite actors belonging to different sectors to seek collaborative projects between them. The National Nutrition Strategies and National Plans of Action on Nutrition that operationalised the nutrition policy would not have been possible, or would have been poorly effective without the emergence of a single grand coalition.

The positive impact of the emergence of a large coalition can be illustrated by the example of the inclusion of Lao PDR in the international Scaling Up Nutrition (SUN) Movement in 2011. SUN is a movement that supports country-led efforts to establish effective systems to address malnutrition. It aims to strengthen the government’s commitment to diminishing malnutrition levels by holding the government accountable for the outcomes of nutrition activities undertaken in the country. To become a member of the SUN Movement, the government is required to establish a recognised multisectoral coalition that is led by the government and includes actors from different sectors. This approach promotes intersectoral collaboration and a collective effort to improve the nutrition status of the population. The SUN Movement currently counts 65 countries and four Indian states among its members. In Lao PDR, the SUN Movement has been an active supporter of the government's efforts to strengthen nutrition policies and initiatives. One of the key ways in which the SUN Movement has contributed to such efforts is by mobilising resources and bringing together diverse stakeholders from different sectors, including government, academia, civil society, the United Nations, donors, and the private sector, to work together towards improving nutrition outcomes [31].

In concrete terms, the operational pillar of the Lao coalition on nutrition consists of the National Nutrition Committee, which was established in 2013, and its secretariat. The National Nutrition Committee is cochaired by a vice-prime minister and the Ministry of Health. An organisation chart with two-headed management aims to provide strong support to the leadership of the minister of health. The members of the National Nutrition Committee include vice-ministers from 12 ministries and representatives of the mass organisations. Four leading agencies are specifically in charge of implementing its activities: the Ministry of Health, Ministry of Education and Sports, Ministry of Planning and Investment, and Ministry of Agriculture and Forestry.

In 2016, Provincial Nutrition Committees were established to promote intersectoral collaborations at the subnational level. The structure was replicated at the district level.

The National Nutrition Committee considered the multisectoral convergence approach, and it issued operational guidelines in 2019. The Lao government uses the convergence approach to encourage and promote common geographic targeting and technical coordination, so that various sectors (primarily health, education, and agriculture) can provide a range of nutrition-specific and nutrition-sensitive services and support in the same geographic locations (districts and villages) to achieve common goals. The convergence approach in action includes support from the World Bank and the European Union’s Programme for Improved Nutrition [32].

In summary, the majority of interviewees (18 of interviewees) claimed that the NNP was the decisive tool to effectively merge the preexisting coalitions. Both national actors (Department of Hygiene and Health Promotion, Nutrition Centre, research institutes, etc.) and external actors (the World Bank, the European Union, the International Fund for Agricultural Development, the World Food Programme, and other development partners) found that the structures and processes put in place through the National Nutrition Policy have encouraged them to develop collaborations between themselves. Above all, the participants all confirmed the feeling that the impact has been positive at all levels ranging from the national level to the community level. Moreover, many respondents (8 respondents) stressed the fact that the changes brought by the policy have made those working in the nutrition world feel that they were part of the efforts deployed by the government to reduce poverty. The expertise gained through intersectoral projects helped to ensure the alignment of projects with the government's priorities and national policies. For many, this was a source of motivation.

*“Nutrition policy pushed sectors to collaborate by developing project that involve sectors such as health, agriculture, and education, at all levels: central, provincial and district levels”.* [P18]

## Discussion

This study aimed to examine the evolution of the coalition of actors involved in the field of nutrition in the Lao PDR before and after the implementation of the NNP, with a focus on the interface between governmental and external actors under the perspective provided by the theory of coalition structuring [15].

Previous studies have suggested that the lack of intersectoral projects that prevailed before the NNP was a determinant of the difficulty of reducing malnutrition in Lao PDR [6, 33]. Before the NNP, the health, agriculture, and education sectors coalitions coexisted and tended to operate largely as single sectors focused on their own issues. There was not enough effort made to exploit the potential synergy between these coalitions. The absence of real collaboration between the concerned sectors was felt by all the respondents to be the main reason why malnutrition—a problem whose determinants are multisectoral—remained an issue despite all the efforts deployed to reduce the problem. The health sector could not effectively and efficiently achieve the objective of reducing malnutrition on its own [10, 34].

This situation changed drastically with the advent of the NNP in 2008. The NNP was the first legally binding document involving all stakeholders in the field of nutrition. The policy designates which ministries and government organisations should participate in the coalition, and which role is expected from each of them. However, the participation of the major stakeholders was not achieved from the outset. Such participation required more than just the formal adoption of the policy in 2008. At the national level, it was not until the advent of the National Nutrition Committee in 2013 that nonhealth sectors at the national level actively joined the coalition. At the provincial level, such committees were implemented in 2016. Thus, the process of integrating the actors into a single coalition took place over several years.

There is literature to argue that intersectoral dialogue requires a unique coalition of multisectoral stakeholders [6]. Indeed, it is known that one of the main obstacles to intersectoral communication is that each sector tends to give more importance to indicators that are of particular interest to them but not considered as much by others [35, 36]. A single coalition provides a platform for understanding each other and, thus, overcoming this obstacle [37].

The main potential force that the government exploited to bring stakeholders into the coalition was the transaction force. Elaborating the National Nutrition Policy has led actors to better realise the benefits that they could gain from exchanging assets with organisations that belong to another sector. Formal meetings between members from different sectors are a forum where specific assets, such as expertise, resources, or the power to authorise action, held by some actors belonging to other sectors can be presented as a potential benefit to other actors [28].

The second force used by the government to build its single coalition involved the control dimension. This control was essentially exercised through the political leadership that was conferred by the government to the Ministry of Health. The official designation of the Ministry of Health as the leader of a multisectoral movement has removed any doubt about the government's commitment to effective leadership in addressing malnutrition in the country. Political leadership is key for garnering support for issues among different actors [38]. The Ministry of Health was henceforth legitimised to elaborate interministry programmes that would be accepted by other ministries. However, this movement was not exceptional. The Lao PDR followed the same path that had been taken by many other developing countries, for example: Bangladesh, Peru and Brazil [37].

Finally, intangible factors were the elements that consolidated the formation of a coalition that already relied heavily on transactions and control. These intangible factors were undoubtedly supported by the political organisation of the country, which is based on a single-party system, and, therefore, the existence of a shared vision of social and health issues in the government apparatus. Intangible factors may also have been enforced by the fact that Lao PDR is a relatively sparsely populated nation and individuals who are involved in various sectors addressing nutrition frequently encounter one another. External actors have to collaborate with government actors from different sectors. The interpersonal links that are often favourable to the functioning of collaborations are often present [39].

Lao PDR has made progress in reducing malnutrition rates since the implementation of the National Nutrition Strategy in 2008. The prevalence of stunting in children under 5 years of age has decreased from 40% in 2006 to 33% in 2017, indicating a significant reduction in the number of children affected by chronic malnutrition [3, 10]. However, the COVID-19 pandemic has presented new challenges to nutrition and food security in the country. The social and economic crisis resulting from the pandemic has posed significant risks to the nutritional health and survival of young children, women, adolescents, and the general population. Access to food, healthcare, education, and nutrition services has been adversely impacted, leading to possible long-term effects on child growth and development, education, and overall human capital development [40].

Almost all Southeast Asian countries have adopted national nutrition plans aiming to strengthen multisectoral approaches to address malnutrition [41, 42]. However, each country faces unique challenges in implementing their nutrition plans and achieving their goals. For example, Indonesia's nutrition plan has encountered difficulties in addressing the high prevalence of stunting and wasting in some regions due to various factors, including poor coordination, inadequate strategies, limited stakeholder engagement, unequal collaboration structures, a shortage of human resources, and budget constraints [43, 44].

The Lao case highlights the importance in this country of promoting government-guaranteed leadership to engage multiple actors, including external actors, to collaborate effectively and achieve a well-defined goal. While this leadership is a complex task that involves multiple sectors, but it is not impossible. As demonstrated by the example described in this work, the emergence of a public policies is a tool that can make such leadership possible.

### Limitations

This study has some limitations. First, semistructured interviews were the main data source of this study. The sample of respondents was obtained primarily based on a reasoned choice approach and the snowball sampling approach, which poses a risk of representative bias [21]. In addition, eight potential participants (e.g., decision-makers and managers) did not respond to the invitation to participate in this study. This lack of response could have prevented us from accessing the entire scope of points of view that are generally looked for in qualitative research [23]. However, these individuals were substituted by others from the same organisations who were sufficiently knowledgeable to speak about what occurred within their organisations before and after the emergence of the NNP. Second, there is a limitation related to the risk of expansiveness bias, which refers to the propensity of respondents to over- or underreport events [45]. The NNP of Lao PDR was implemented in 2008. We recognise that several participants might have had some difficulties recalling the events and sometimes hesitated in answering, especially regarding the situation before the emergence of the policy. Finally, we acknowledge that it is possible that some of the respondents may have personal or vested interests in promoting the effectiveness of the NNP, particularly those who are directly involved in the development and implementation of the NNP. To mitigate this bias, this study included both governmental and external participants, including those who were involved prior to the emergence of the NNP in 2008 and those who were not. During the interview, we encouraged the participants to provide honest and objective answers. Moreover, our study does not aim to promote or endorse the effectiveness of the NNP but rather to provide the perceptions of key stakeholders regarding the emergence of the NNP. There is a need for further research to evaluate the strengths and weaknesses and the actual impact of the NNP or National Nutrition Strategy and Plan of Action on nutrition outcomes in Lao PDR.

## Conclusion

Combating malnutrition is a government priority in the Lao PDR. This study showed that there has been a significant evolution in the malnutrition actor coalition landscape in Lao PDR following the emergence of the National Nutrition Policy. The policy has provided a framework for coordinated action among various stakeholders, leading to improved collaboration and strengthening existing ones. The finding suggests that a public policy, such as the National Nutrition Policy, was an effective or even necessary tool for formalising and enabling intersectoral activities that more effectively address a complex problem.

## Data Availability

All data analysed during this study are included in this article.

## Abbreviations

FAO: Food and Agriculture Organisation
Lao PDR: Lao People’s Democratic Republic
NGOs: Nongovernmental organisations
NNP: National Nutrition Policy
SUN: Scaling Up Nutrition
UN: United Nations
UNICEF: United Nations Children's Fund
WHO: World Health Organisation
